# Supplementary material for: Non-Apoptotic Caspase-3 Activation Mediates Early Synaptic Dysfunction of Indirect Pathway Neurons in the Parkinsonian Striatum
Source: Int J Mol Sci. 2022 May 13;23(10):5470. doi: 10.3390/ijms23105470 (PMC9141690; doi:10.3390/ijms23105470)
Supplement: Supplementary file 1 [file ijms-23-05470-s001.zip › ijms-1669508-supplementary.pdf]

Supplementary Material

Supplementary Figure S1

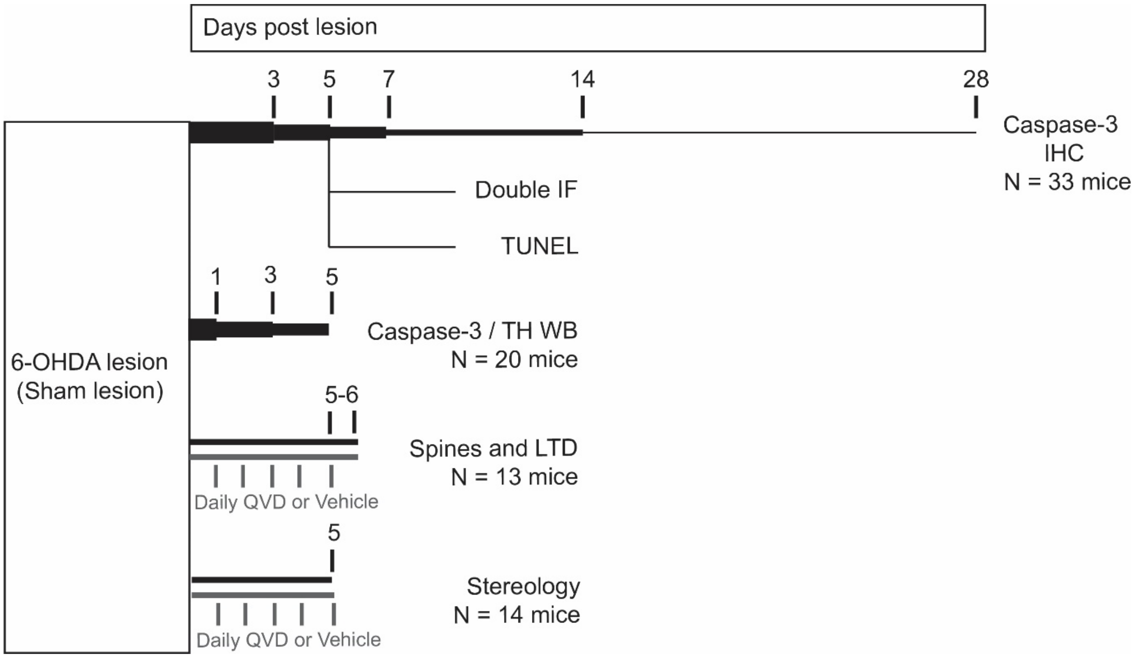

Figure S1. Experimental layout. From top to bottom, the different arms show the different experimental groups the mice were divided into after 6-OHDA or Sham surgery. The different time points within the experimental arms are indicated by the numbers. Daily treatments are marked for the lower two arms. The type of experiment and number of mice used is given at the right end of every arm. A total of 80 mice was used in this study.

Supplementary Table S1. Details of statistical tests.

Figure		Test		P value	Comment
1	G	One-way ANOVA	F (5, 27) = 4.625	0.0035	Caspase-3 increase over time (IHC)
		Bonferroni's multiple comparison test (vs. Sham)		0.038	Day 3
				0.0022	Day 5
				0.5429	Day 7
				>0.9999	Day 14
				>0.9999	Day 28
2	A	One-way ANOVA	F (2, 17) = 106.7	<0.0001	TH decline over time
		Bonferroni's multiple comparison test		<0.0001	Day 1 vs. Day 3
				<0.0001	Day 1 vs. Day 5
				0.3108	Day 3 vs. Day 5
	B	One-way ANOVA	F (2, 17) = 4.321	0.0316	Caspase-3 increase over time (IHC)
		Bonferroni's multiple comparison test		>0.999	Day 1 vs. Day 3
				0.0449	Day 1 vs. Day 5
				0.0947	Day 3 vs. Day 5
3	A	One-way ANOVA	F (2, 29) = 19.43	<0.0001	Dendritic spines
		Bonferroni's multiple comparison test		<0.0001	Sham vs. 6-OHDA
				0.5077	Sham vs. 6-OHDA + QVD
				<0.0001	6-OHDA vs. 6-OHDA + QVD
	F	One-way ANOVA	F (2, 17) = 24.44	<0.0001	EPSC post HFS
		Bonferroni's multiple comparison test		0.0002	Sham vs. 6-OHDA
				0.7627	Sham vs. 6-OHDA + QVD
				<0.0001	6-OHDA vs. 6-OHDA + QVD
	G	Paired t-test	t=3.609, df=5	0.0154	Sham
		Paired t-test	t=0.6847, df=7	0.5155	6-OHDA
		Paired t-test	t=3.507, df=5	0.0171	6-OHDA + QVD
4	C	One-way ANOVA	F (2, 11) = 2239	<0.0001	Striatal TH
		Bonferroni's multiple comparison test		<0.0001	Sham vs. 6-OHDA
				<0.0001	Sham vs. 6-OHDA + QVD
				>0.9999	6-OHDA vs. 6-OHDA + QVD
	D	One-way ANOVA	F (2, 11) = 6.018	0.0172	Nigral DA neurons
		Bonferroni's multiple comparison test		0.0430	Sham vs. 6-OHDA
				0.0336	Sham vs. 6-OHDA + QVD
				>0.9999	6-OHDA vs. 6-OHDA + QVD
